# The largest amber-preserved flower revisited

**DOI:** 10.1038/s41598-022-24549-z

**Published:** 2023-01-12

**Authors:** Eva-Maria Sadowski, Christa-Charlotte Hofmann

**Affiliations:** 1 Museum für Naturkunde, Leibniz Institute for Evolution and Biodiversity Science, Invalidenstraße 43, 10115 Berlin, Germany; 2 Institut für Paläontologie, University of Vienna, Josef-Holaubek Platz 2 (UZA 2), 1090 Wien, Austria

**Keywords:** Plant evolution, Palaeontology, Taxonomy

## Abstract

Amber exquisitely preserves the delicate organs of fossil flowers for millions of years. However, flower inclusions can be rare and usually do not exceed 10 mm in size. Here we report an exceptionally large flower from late Eocene Baltic amber, measuring 28 mm across, which is about three times as large as most floral inclusions. This fossil was described over 150 years ago as *Stewartia kowalewskii* (Theaceae) and has never been revised. The analysis of pollen extracted from the anthers of the flower inclusion, however, revealed strong affinities to Asian species of *Symplocos* (Symplocaceae), prompting the new combination *Symplocos kowalewskii* comb. nov. et emend. This fossil represents the first record of Symplocaceae from Baltic amber and supports affinities of its flora to evergreen broadleaved and mixed mesophytic forests of present-day East and Southeast Asia. The rarity of such large-sized flower inclusions is likely due to the size of the resin outpouring and its properties, which might affect the embedding of plant organs.

## Introduction

Amber preserves organisms three-dimensionally and with great fidelity, including arthropods, fungi, bryophytes, lichens, as well as minute inclusions of seed plants, such as leaves, flowers, catkins and pollen^1–7^. These inclusions are otherwise rare from the fossil record and therefore can yield new insights into palaeoecosystems and their biota ranging from the Triassic up to the Cenozoic^8^.

Whereas inclusions of arthropods are most abundant, plant inclusions are generally rare. Only 1–3% of all inclusions from late Eocene Baltic amber are of botanical origin^9,10^. However, the botanical inclusions that are present are valuable for understanding the evolution of plant lineages, their palaeobiogeographic history and the amber source area, including habitats, plant diversity and the palaeoclimate^3,7,11–17^. Although their exquisite preservation often allows assignment to genus or even species, most botanical amber inclusions are small in size. For example, inclusions of detached flowers from Baltic amber mostly range between a few millimeters and about 15 mm in size^7,18,19^ (Supplementary Table S1), which is also the case for most flower inclusions from other amber deposits (Supplementary Table S1 and references therein). Here, we focus on a corolla inclusion with attached stamens from late Eocene Baltic amber measuring 28 mm in diameter. As such, it is the largest floral inclusion from all ambers known. The analysis of gross morphology and in-situ pollen extracted from the inclusion justifies its assignment to *Symplocos* (Symplocaceae, sweetleaf family), thus making the fossil the first record of this family from Baltic amber.

## Results

### Systematics

Order: Ericales Dumortier

Family: Symplocaceae Desf.

Genus: *Symplocos* Jacq.

*Symplocos kowalewskii* (Casp.) Sadowski et Hofmann comb. nov. et emend.

**Basionym:** *Stewartia kowalewskii* Casp. 1872, p. 17 [no figure].

**Figure 1:**
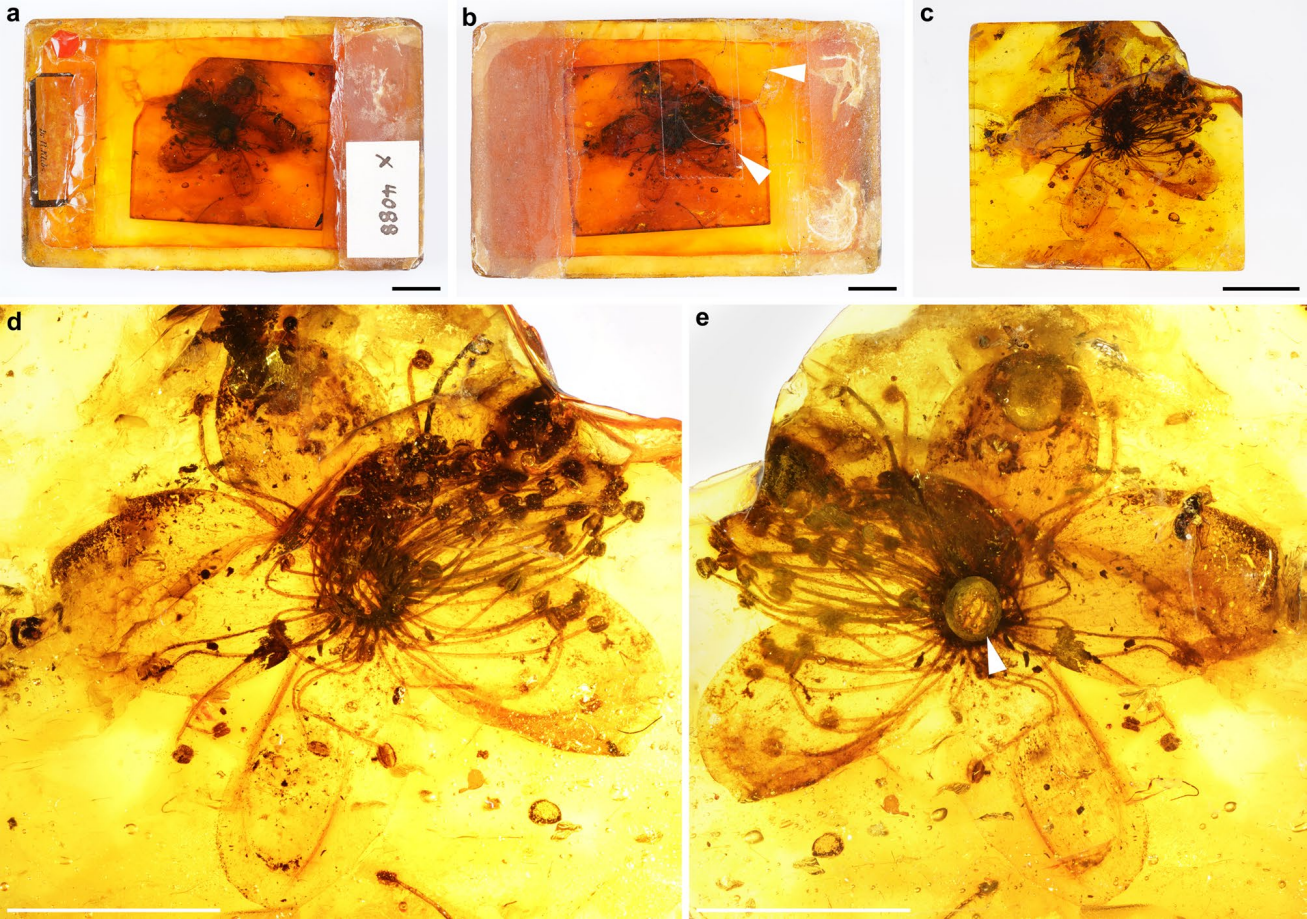
Flower inclusion of *Symplocos kowalewskii* comb. nov. et emend. (Symplocaceae; X4088) from late Eocene Baltic amber. (**a**,**b**) Overview of the historic preparation of the amber specimen before restauration, showing fissures (**b**, upper arrowhead) and discoloration. The fractured cover slip had been fixed with tape (**b**, lower arrowhead). (**c**) Overview of the amber specimen after extraction from the glass chamber. (**d**,**e**) Flower from the upper (**d**) and lower side (**e**) after preparation. Note the fused staminate ring on the underside (**e**, arrowhead). Scale bars 1 cm in (**a**‒**e**).

**Figure 2:**
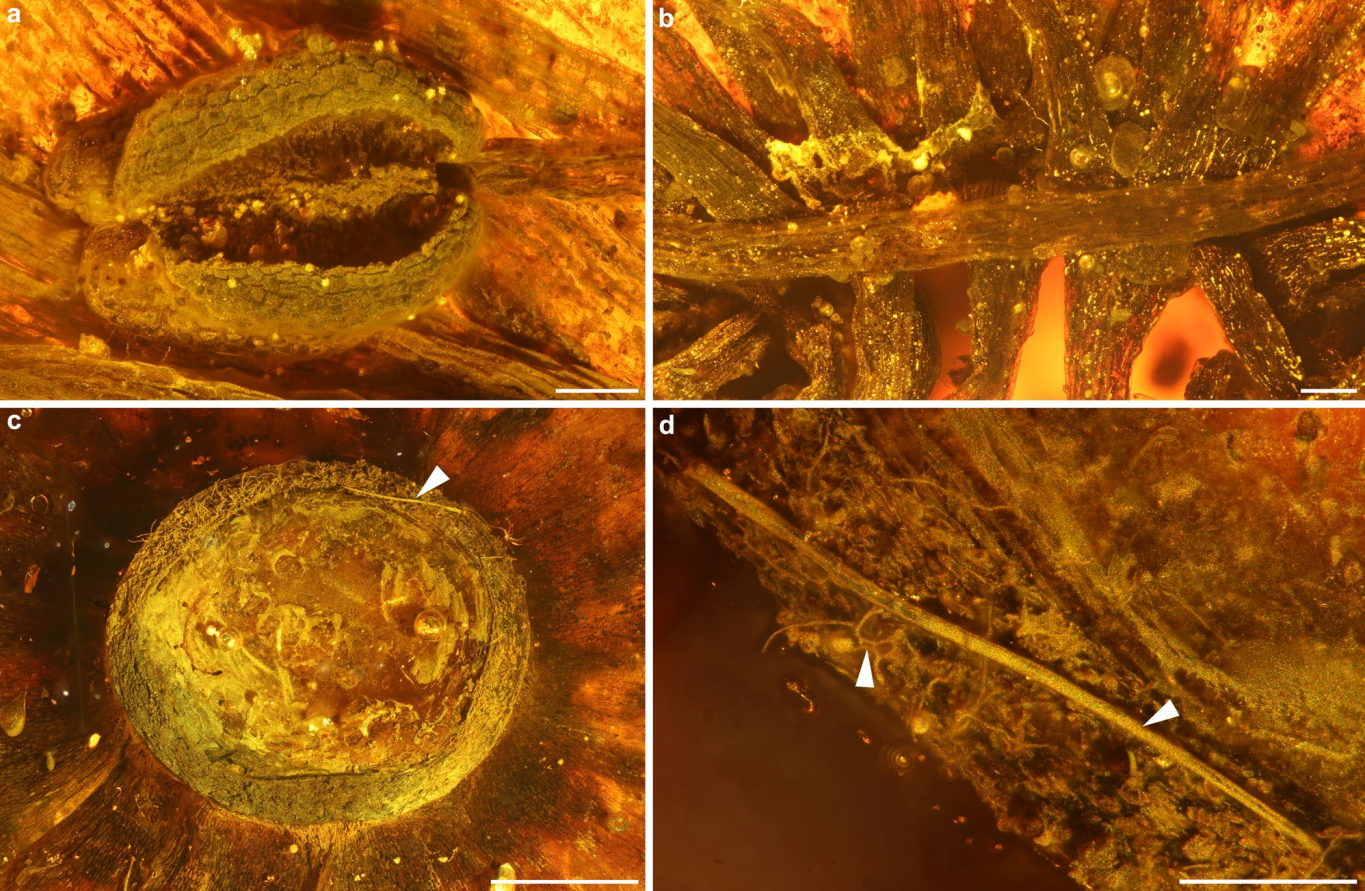
Details of *Symplocos kowalewskii* comb. nov. et emend. (Symplocaceae; X4088) from late Eocene Baltic amber. (**a**) Anther. (**b**) Basally fused and broadened filaments, arranged in three rows and forming a fused ring. (**c**) Underside of the fused staminate ring with trichomes (arrowhead). (**d**) Simple, long, acute trichome (right arrowhead), magnified from (**c**); left arrowhead indicates fungal hyphae. Scale bars 200 µm in (**a**), (**b**), (**d**); 1 mm in (**c**).

**Figure 3:**
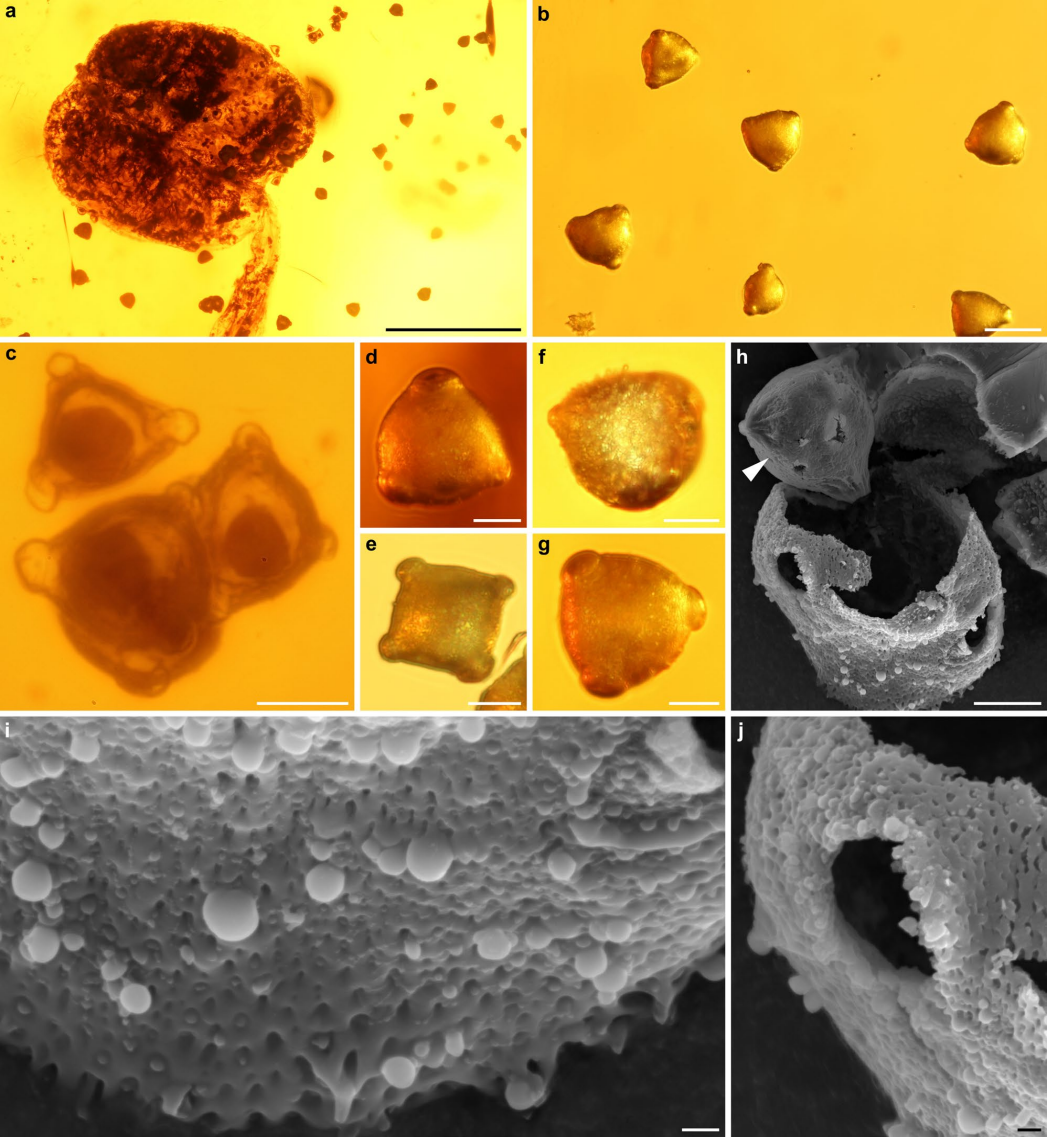
Pollen extracted from anthers and the surrounding amber of *Symplocos kowalewskii* comb. nov. et emend. (Symplocaceae; X4088) from late Eocene Baltic amber. (**a**) Anther, releasing pollen. (**b‒g**) Pollen under LM; note the protrusions from the apertures (**c**), which were likely caused by the excreted internal cell content. (**h**) Pollen under electron microprobe, with exposed intine (arrowhead). (**i**,**j**) Pollen under electron microprobe, showing perforate to microreticulate ornamentation with few supratectal blunt echini. Note the fused verrucae at the margo of the ectoaperture, forming a rim-like structure (**j**). Scale bars 500 µm in (**a**); 50 µm in (**b**); 20 µm in (**c**–**g**); 10 µm in (**h**), 1 µm in (**i**,**j**).

**Holotype:** X4088, figured in Figs. 1–3. Repository: Federal Institute for Geosciences and Natural Resources

(Bundesanstalt für Geowissenschaften und Rohstoffe, BGR), Berlin, Germany.

**Plant Fossil Names Registry Number**: PFN003014.

### Additional references

1886 *Stuartia kowalewskii* Casp.—Conwentz, p. 63 [no figure].

1890 *Stuartia kowalewskii* Casp.—Schenk, p. 517 [no figure].

1921 *Stewartia* L.—Gothan, p. 391 [no figure].

1929 *Stuartia kowalewskii* Casp.—Gothan, p. 114, Abb. 1 and figure on p. 128.

1948 *Stuartia* L.—Gothan, p. 20, Abb. 9a, a1.

1954 *Stuartia* L.—Gothan and Weyland, p. 417 [no figure].

1957 *Stuartia kowalewskii*—Kirchheimer, p. 584 [no figure].

1964 *Stuartia* L.—Gothan and Weyland, p. 455 [no figure].

1970 *Stuartia kowalewskii* Casp.—Rüffle and Helms, p. 247, pl. 2, fig. 2.

2000 *Stuartia kowalewskii* Casp.—Rüffle and Litke, p. 451, pl. II, fig. 1.

### Emended diagnosis

Petals fused at the base into a ring-like structure. Outer surface of ring covered with few long simple trichomes. Stamens numerous, almost as long as petals, arranged in three consecutive rows. Pollen tricolporate, occasionally tetracolporate, with short colpi (brevicolpate) and conspicuous vestibulate apertures, exine is tectate, perforate and scabrate to verrucate (light microscopy; LM), and perforate to microreticulate on short columellae with occasionally occurring supratectal verrucae and echini (scanning electron microscopy; SEM).

### Description

*Corolla:* 25–28 mm in diameter; petals five, fused at base (gamopetalous), linguiform to obovate, 7.2–9.3 × 11–13 mm, membranaceous, glabrous (Fig. 1a–e); at base forming a ring-like structure (Figs. 1e, 2c), 2.8 mm in diameter × 1 mm long, rim of ring 0.3 mm wide, covered with few trichomes (Fig. 2d). Trichomes simple, unbranched, acute, up to 880 µm long × 20 µm wide (Fig. 2d). *Receptacle, calyx, and gynoecium:* not preserved. *Androecium:* Stamens arranged in three rows, fused to base of petals (Fig. 2b), numerous, > 74 (Fig. 1c–e); filaments flattened, (5.3–) 8.22 (–11) mm long × (149–) 220 (–460) µm wide (middle part measured), base dilated (Fig. 2b), 240–260 µm wide, apex constricted (Fig. 2a); anthers with two thecae, basifixed, subglobose, (832–) 1073 (–1290) µm long × (832–) 911 (–1040) µm wide, base cordate, apex notched (Figs. 2a, 3a). *Pollen:* tricolporate to tetracolporate, with short colpi (= brevicolpate; Fig. 3b,c,e), oblate to subspheroidal with typical vestibulate apertures, outline in polar view ranges from triangular, triangular convex to circular (Fig. 3c–g), equatorial diameter 30–70 µm; the ratio of the length of the polar axes and colpi is variable ranging from 2.3 to 3.2 (N = 6); thickness of ektexine (tectum, columellae and footlayer) ca. 0.6 µm, tectum and columellae 0.2–0.3 µm thick and in apertural region ca. twice as thick (LM). In LM: ektexine seems tectate and shows perforate, scabrate to loosely verrucate sculpture (Fig. 3f). In SEM: ektexine sculpture is perforate to microreticulate with occasionally occurring supratectal verrucae [diameter 0.3–0.8 (–1.5 µm)] and few supratectal blunt echini (Fig. 3h–j). Colpus length 8–12 µm long, colpus width 3–4 µm in the equator area, colpus apex weakly pointed; supratectal verrucae often fused at margo of ectoaperture into a rim-like structure (Fig. 3j); colpus membrane is microverrucate; endoporus ca. 5–6 µm high (width not discernable, but endoaperture appears to be more lalongate in outline).

### Remarks

The fossil was first published as *Stewartia kowalewskii* Casp. (Theaceae; also occasionally spelled *Stuartia*^20^), but not figured and only briefly described as a well preserved pentamerous corolla of 28 mm in diameter with attached stamens^18,21^. Since then, the flower inclusion was frequently mentioned by various authors and occasionally figured over the last decades^22–29^. However, it was never documented in detail nor its identification thoroughly assessed. Kirchheimer^30^ considered the fossil as similar to *Stewartia* I.Lawson but thought that the corolla did not provide sufficient evidence to demonstrate affinities to *Stewartia*. Affinities to the Theaceae, specifically the Camellioidae, were further suggested^31,32^ but never unambiguously proven. Indeed, the inclusion resembles members of the Camellioideae (including *Stewartia*) in, for example, the basally connate and numerous (uncountable) stamens arranged in rows; the basifixed anthers (basifixed in some *Camellia* L. species, but dorsifixed in *Stewartia*) which lack an apical prolongation of the connective; the length of filaments, which are nearly as long as the petals; and the basally fused corolla^32–36^ (Table 1). According to Tsou^37,38^, the only diagnostic character of the Camellioideae is the presence of pseudopollen in the connective of the anthers. We could not detect any pseudopollen in the anthers of the amber specimen. However, we are aware that despite the exquisite preservation of the amber specimen, the presence of such pseudopollen would be difficult to assess because it is rather small and inserted into the connective.

**Table 1:** Comparison of *Symplocos kowalewskii* comb. nov. et emend. from late Eocene Baltic amber to fossil and extant flowers of the Symplocaceae and Theaceae. Information that was not available is indicated with ‒.

Genus	*Symplocos kowalewskii*	*Symplocos bureauana*	*Symplocos subspicata*	*Symplocos* subg. *Symplocos*	*Stewartia* (Theaceae)
Distribution	Samland Peninsula (Kalinigrand, Russia)	Sézanne, Châlons (Marne, France); Wimmelburg near Eisleben (Saxony-Anhalt, Germany)	Wimmelburg near Eisleben (Saxony-Anhalt, Germany)	Americas, eastern Asia, Australasia	China, Japan, Korea, South Eastern United States
Age	Late Eocene (Priabonian)	Early Eocene, early Oligocene	Early Eocene, early Oligocene	Extant	Extant
Flower size (mm)	25‒28	(7‒) 8 (‒1)	‒	(3‒) 13 (‒50)	20‒50
**Petals**	5; basally fused forming a low ring	5; short tube	5; basally fused, forming a short, pentagonal tube	(3‒) 5 (‒15); basally fused or connate beyond base	5; basally slightly connate, imbricate
Shape	Obovate, rounded	Ovate-oblong, lanceolate, acute	Linguiform	Oblong	Obovate
**Stamens**	> 74	18	25‒30	(4‒15‒) 40‒100	Numerous
Arrangement	Arranged in three rows; basally adnate to petals	In 5 groups, each with 3 stamens, alternating with petals, arranged in one row; non-fused	In groups, each with 5–6 stamens, antepetalous, arranged in one row, basally fused with the tube	Uniseriate or 2–4-seriate; basally adnate to petals	Adnate to petals or free
Filaments	Non-monadelphous, flattened, broadening towards base, apically constricted; glabrous	Non-monadelphous, no broadening towards base	Non-monadelphous, wider towards base	(Non-)monadelphous; terete or tangentially flattened; apically constricted or not; glabrous	Basally connate, forming a tube
Anthers	Apically notched, base cordate; subglobose, basifixed	Apically rounded, base cordate	Globose	Basifixed	Dorsifixed, versatile
Pseudo pollen	Absent	‒	‒	Absent	Present

Data from^29,32,33,35–38,40,55–57,74,76,96,97^. Flower diameter of *S. bureauana* measured from the holotype MNHN.F-2170.2.

The extracted pollen of the fossil shows distinct features of *Symplocos* Jacq. (Symplocaceae) as it exhibits: tricolporate apertures with short colpi (polar axes/colpus length ratio), oblate to subspheroidal shape with a triangular to circular outline in polar view and conspicuous vestibulum. Tectum sculpture and ornamentation is variable: densely verrucate, rugulate to verrucate, a combination of rugulate to microreticulate, perforate, microverrucate, and microreticulate with or without supratectal ornamentation^39^. Additionally, the combination of gross morphological characters is also indicative for *Symplocos* (Symplocaceae), including gamopetalous corolla, androecium adnate to corolla, stamen non-monadelphous and numerous in three consecutive series, filaments thin and constricted at apex, anthers subglobose with two thecae^40^.

According to Fritsch et al.^40^, the Symplocaceae encompass two genera, *Symplocos* and *Cordyloblaste* Hensch. Ex Moritzi. However, in *Cordyloblaste*, the stamens are fused (monadelphous), androecium adnation to the corolla is roughly to the midpoint of the corolla, and petals are coriaceous^40^. The pollen of *Symplocos* and *Cordyloblaste* share some similarities but can be distinguished by the supratectal ornamentation, which is present in *Symplocos* and *S. kowalewskii*, but lacking in *Cordyloblaste*^40–42^.

*Symplocos* subgenus *Palura* (G.Don) P.W.Fritsch (with only one species, *S. paniculata* Miq.) and *Symplocos* subgenus *Symplocos* are distinguished by characters that are mainly not preserved in the fossil, e.g., the numbers of carpels of the gynoecium. However, filaments of *S. paniculata* are terete and not constricted apically^40^, whereas they are flattened in the amber specimen and taper towards the anthers. Moreover, in contrast to the amber specimen, the pollen of *S. paniculata* is rather small (26–28 µm in diameter) and has a triangular to concave triangular outline in polar view. Furthermore, the pollen of *S. paniculata* is unique in the rugulate to microreticulate sculpture with perforations and fossulae in between the rugulae, producing a bireticulated pattern. Also as opposed to the amber specimen, pollen of *S. paniculata* has no supratectal ornamentation^43,44^.

Therefore, the amber specimen is more closely affiliated with *Symplocos* subgenus *Symplocos*. As based on phylogenetic analysis, this subgenus is divided into taxa (corresponding to clades), including *Symplocos* sections *Barberina* A.DC., *Lodhra* G.Don and *Symplocos.* The latter is divided into series *Symplocos* and *Urbaniocharis* (Brand) P.W.Fritsch^40^. The fossil can be excluded from sect. *Symplocos* based on the combination of its large size (thus excluding series *Urbaniocharis*, the species of which have corollas < 10 mm long^45^), the non-monadelphous stamens (in series *Symplocos*, stamens are connate roughly halfway), and the androecial adnation merely at the base of the corolla (androecium is adnate about halfway to the corolla in series *Symplocos*). Moreover, within series *Symplocos*, the informal group (clade) “*Neosymplocos*” is distinguished from the fossil by its pubescent filaments^40^.

The remaining sections *Barberina* and *Lohdra* can only be effectively compared to the amber fossil on the basis of pollen morphology. About 86 extant *Symplocos* pollen species have been documented in the literature^41,42,44,46,47^. However, the documentation of sculpture variation of extant as well as fossil *Symplocos* pollen with SEM is incomplete because most pollen images are depicted only with light microscopy^48^. In comparing the available extant *Symplocos* pollen types with those from *S. kowalewskii*, only a few Asian species resemble the amber specimen in shape, size, outline and ektexine sculpture and supratectal ornamentation, namely *S. obtusa* Wall.*, S. pergracilis* (Nakai) Yamazaki*, S. tanakae* Matsamura, and to a lesser extent *S. pseudobarberina* Gontscharow (all of *S.* section *Lodhra*). These species are all characterized by a perforate to microreticulate tectum and supratectal verrucae and occasional supratectal echini^40,42^, which is somewhat similar to *S. kowalewskii.* However, the density, number and sizes of these supratectal elements differ from those in *S. kowalewskii* and vary considerably among the named extant species (quite dense in *S. tanakae,* larger and more loose or regularly distributed in the other species).

In section *Barberina*, some resemblance occurs in the tectum of *S. variabilis*^49^; however, the overall shape and the rounded apex of the colpus differ from the states of *S. kowalewskii.*

Among the fossil record, pollen of *S. kowalewskii* resembles two fossil *Symplocos* pollen types from the early Oligocene Haselbach locality (Germany^43^, *Symplocos* sp. 2 and sp. 8) in being microreticulate to foveolate or perforate with supratectal verrucae and baculae. As in *S. kowalewskii*, these pollen types bear similarities to the extant Asian species *S. obtusa, S. pergracilis, S. tanakae* and *S. pseudobarberina.*

All in all, the flower and pollen morphology of the amber inclusion is indicative enough to justify its assignment to *Symplocos* subgenus *Symplocos* with the new combination *Symplocos kowalewskii* (Casp.) Sadowski et Hofmann comb. nov. et emend*.* Based on the available literature, comparisons of *S. kowalewskii* with extant and fossil *Symplocos* indicates affinities to Asian taxa, especially to some species in *S.* section *Lodhra.* However, future studies that comprehensively document pollen of Symplocaceae are necessary to elucidate distinct affinities of *S. kowalewskii* to extinct and modern lineages of the family.

## Discussion

### Fossil record of *Symplocos*

The oldest fossil record of Symplocaceae is pollen from the Maastrichtian of California, which is, however, regarded as doubtful^48,50^. In contrast, the oldest unambiguous *Symplocos* fossils are fruits from the early Eocene of the Fisher/Sullivan site in Virginia (United States)^51^ and from the lower Eocene of Central and Western Europe (^52^, and references therein). Fruits of *Symplocos* are drupes with a lignified endocarp, which is very resistant to decay. Therefore, fossilized *Symplocos* endocarps abundantly occur in the fossil record^52^. Previously, 13 fossil *Symplocos* species of fruits have been confirmed from the European Neogene^52,53^. Three fossil species of fruits (*S. headonensis* Chandler*, S. lakensis* Chandler*, S. trilocularis* Reid et Chandler) are restricted to the European Paleogene and still lack confirmation as to whether they are really separate taxonomic entities^52^. There is no fossil flower of *Symplocos* known to be associated with endocarps and therefore possible affinities of *S. kowalewskii* to the known fossil species are difficult to assess. Several fossil endocarps of *Symplocos* resemble those of extant species such as *S. anomala* Brand*, S. foliosa* Wight*, S. lucida* (Thunb.) Siebold et Zucc. sensu Nooteboom^35^*, S. microphylla* Wight*, S. ramosissima* Wall. ex G. Don*, S. tinctoria* (L.) L’Hér. and *S.* section *Palura* [^52^ = subgenus *Palura*]*.* However, the pollen morphology of the named extant species is very different from pollen of *S. kowalewskii*. As per Mai and Martinetto^52^, fruits of lower Miocene to upper Pliocene *Symplocos schereri* Kirchheimer are similar to extant *S. tanakae*. Pollen of *S. tanakae* resembles that of *S. kowalewskii* (see above for details) from which one could infer a possible link between *S. schereri, S. tanakae* and *S. kowalewskii*. However, there are no fossil flowers of *S. schereri,* which would allow a more detailed comparison with the amber fossil. Moreover, extant *S. tanakae* differs from *S. kowalewskii* in having pentadelphous stamens and a corolla of 6‒7.5 mm length^54^.

Fossil flowers of *Symplocos* are rare, with only two species confirmed^55^: *Symplocos bureauana* Sap. (lower Eocene, Sézanne, France; Eocene of Wimmelburg near Eisleben, Germany^56,57^) and *Symplocos subspicata* Friederich (Eocene of Wimmelburg near Eisleben, Germany^57^). Imprints of calyces named *Symplocos myosotis* (Unger) Weyland (upper Oligocene, Rott near Siegburg, Germany^58,59^) and *Symplocos parschlugiana* Unger (middle Miocene, Parschlug, Austria^60^) do not provide enough evidence to confirm their affiliations with Symplocaceae^55^. An additional fossil report with suggested affinities to flowers of *Symplocos* is *Antholithus amoenus* Lesq. from the Green River group in Florissant (Colorado, United States^29,61^); this fossil, however, does not show enough similarities to *Symplocos* and is therefore doubtful^55^.

In *Symplocos bureauana* and *S. subspicata*, the gynoecium was not preserved and pollen extraction from the fossils was unsuccessful^55^. In comparing both species with *S. kowalewskii* (Table 1), they share the basally fused petals which form a very short tube (1 mm for *S. kowalewskii*; about 0.02‒0.04 mm for *S. bureauana*), the number and shape of petals, the stamens being shorter than the petals, and the size of the anthers. However, *S. kowalewskii* differs from both fossils in having a larger corolla (25‒28 mm in *S. kowalewskii*; up to 10 mm diameter in *S. bureauana*), the greater number of stamens (> 74, versus 18 in *S. bureauana* and 25–30 in *S. subspicata*), the length of the filaments (5.3‒11 mm in *S. kowalewskii*; 1.3‒1.5 mm in *S. bureauana*) and the arrangement of stamens in three rows in *S. kowalewskii* (versus one row in both *S. bureauana* and *S. subspicata*^29,55,57^, Table 1). Thus, features of *S. kowalewskii* clearly differentiate it from other fossil taxa, justifying its treatment as a distinct species. Among the numerous flower inclusions from Baltic amber^18^, none shows the same set of indicative features as *S. kowalewskii*. Thus, *S. kowalewskii* is the first fossil record of this genus and of the Symplocaceae from Baltic amber.

### Palaeoecological implications

Symplocaceae from the early Eocene flourished in paratropical forests with deciduous and evergreen taxa and multilayered canopies (e.g. early Ypresian, Fisher/Sullivan site, Virginia, United States^51,62^). In younger fossil floras, Symplocaceae also dominated forested areas (e.g. in Miocene of Vogelberg/Salzhausen, Germany^55^) or grew in the understory of lowland hinterland forests, mixed with conifers and angiosperms (middle Miocene, Lavantal Basin, Austria^48^; late early Miocene, Wiesa, Germany^53,63^). In the early Oligocene Haselbach horizon (Leipzig Embayment, Germany^64^), *Symplocos* species were one of the main constituents of mixed mesophytic forests, but also occurred in *Quasisequoia* swamp forests^65^. Most species of extant Symplocaceae are evergreen shrubs and trees that grow from 500 up to 4000 m elevation of tropical zones, being most abundant in mountain forests of 2500–3500 m elevation^36^. The fossil and extant occurrences of Symplocaceae indicate that the family thrives in humid mixed-mesophytic forests in warm-temperate to subtropical climates, whereas arid regions are avoided^31,44,48,66–68^.

This agrees with the most recent analyses of the Baltic amber source area, the so-called Baltic amber forest, where humid and warm-temperate conditions likely prevailed. Furthermore, assessment of inclusions of hyperdiverse Fagaceae and conifers, as well as fungi and lichens, show that the Baltic amber forest was heterogeneous, including coastal swamps, bogs, riparian forests and mixed conifer-angiosperm forests intermingled with open areas^7,15,16,69–72^. As indicated by the fossil record of Symplocaceae, *Symplocos kowalewskii* was likely part of the forested habitats in the Baltic amber source area. As known from the Oligocene Haselbach flora, it is also possible that *S. kowalewskii* was associated with *Quasiseuqoia* swamps, since this conifer has recently been confirmed from Baltic amber^15,16^.

### Palaeobiogeographical links

Extant Symplocaceae are disjunct between the Americas and Asia, ranging from South Brazil to the Southeastern United States and from the Deccan Peninsula in India to northern China and Japan, reaching New South Wales in Australia and Fiji in the Pacific^35,66^. Symplocaceae possibly originated in Europe about 52 Ma and then dispersed to North America between 52 and 38 Ma, from where several lineages migrated south^66^. Not before the Pliocene, Symplocaceae dispersed from Europe to eastern Asia, as it was indicated by its macrofossil record^66,68^. However, recently discovered fossil pollen of *Symplocos* subgen. *Palura* from the middle Eocene of Hainan^44^ and *Symplocos* pollen from the Paleogene of the far East of Russia (named “*Proteacidites*”^73^) have challenged notions about the Paleogene distribution of Symplocaceae and indicate that the family was already present in Asia by that time.

As discussed above, *Symplocos kowalewskii* and its pollen shows the most similarities to extant species of Asia. *Symplocos obtusa* is found in South India and Ceylon (at elevations of 1800–2400 m^33,35,40^), whereas *S. pseudobarberina* grows in Southeast China (at elevations of 1000 m, from Yunnan to Fujian), Cambodia and Vietnam^74^. *Symplocos tanakae* and *S. pergracilis* are endemic to the Shikoku and Kyushu islands (*S. tanakae*), as well as Bonin Islands (*S. pergracilis*) of Japan^40,54^. Among fossil *Symplocos* pollen, *S. kowalewskii* is similar to pollen types of the Oligocene Haselbach locality, which also show Asian affinities^43^, underlining the close link of Paleogene *Symplocos* from Europe to extant Asia.

Hence, *S. kowalewskii* substantiates the affinities of the Baltic amber source area to East and Southeast Asia. This link has been previously supported by the analysis of Fagaceae and conifer inclusions. They encompassed several genera that are common or restricted to East and Southeast Asia today, such as *Cathaya* Chun et Kuang*, Nothotsuga* Hu ex C.N.Page*, Cephalotaxus* Siebold et Zucc. ex Endl.*, Cryptomeria* D.Don and *Castanopsis* (D.Don) Spach^7,15,16,19^. Among extant endemic plants from East Asia, some have an extensive fossil record in the Northern Hemisphere, where they may have originated and spread. Subsequent climatic cooling and glaciation in the Northern Hemisphere caused these taxa to become extirpated there and restricted to East Asian refugia^75^.

### Flower size and preservation of ***Symplocos kowalewskii***

Among extant Symplocaceae, corollas generally range in size between 3 and 13 mm^33,35^, but also can become larger^76^. Flower size is apparently without consistent taxonomic significance^40^. Nonetheless, corolla size and the degree of petal fusion can be an indicator of different pollination syndromes. For example, the androecium of flowers in most of the species of Neotropical *Symplocos* sect. *Symplocos* is distally more adnate to the tubular-shaped corolla. This is interpreted as adaptation to pollination by long-tongued bees and long-billed hummingbirds that are able to reach the copious nectar^40,77^. In contrast, most Asian species of *Symplocos* are thought to be generally insect pollinated, which would explain why their corollas and androecia are lesser fused than in Neotropical *Symplocos* sect. *Symplocos*^40^. The corolla of *S. kowalewskii* is only basally fused, as is the case for many extant insect-pollinated Symplocaceae. An additional indication for insect pollination of *S. kowalewskii* might be the unusually large corolla that likely served as attraction to insect pollinators^78^.

The exceptional preservation of amber inclusions like *S. kowalewskii* is possibly caused by the biocidal properties of the embedding resin, which inhibits degradation processes^79,80^. In many plant inclusions, internal and external structures are three-dimensionally preserved^13^, similar to mummifications^16^; however, it is unknown as to which processes are involved in the preservation of plants in amber. In contrast, the taphonomy of animal inclusions has been studied in detail, showing numerous factors that control their preservation, such as size of the organism, resin viscosity and stickiness^80–83^. Some of these factors could be similarly important for plant inclusions. Depending on the resin surface tension and viscosity, smaller plant organs are likely more easily retained than larger ones. Based on the known plant inclusions, it is evident that there is a size limitation in amber (Supplementary Table S1). This is probably also related to the size of the resin trap; in Baltic amber only 4.7% of the mined pieces are > 32 mm in size whereas more than 40% measure < 18 mm^84^, showing that larger pieces, as in this study, are rare. In considering the overall scarcity of plant inclusions^9,10^, as well as the taphonomical biases, amber inclusions like *Symplocos kowalewskii* are unique in preservation and size.

Additionally, the flower of *S. kowalewskii* was trapped by the resin during anthesis, providing enough mature pollen for extraction, which is also a rather rare condition in flower inclusions^85^. The amber pollen of *S. kowalewskii* exhibits exceptional preservation as well; for example, SEM analyses revealed preservation of the pollen wall, including the intine (Fig. 3h), which is normally lacking after preparing conventional palynological material^86^. Furthermore, the well-preserved details of the pollen ornamentation facilitated the identification of the flower, which shows the great benefit of pollen extraction, as well as the necessity of SEM analyses.

Some pollen of the amber specimen show protrusion-like structures at the apertures (Fig. 3c), which likely were caused by the excretion of cellular content. This is somewhat similar to the effect of acetolysis where the cellular content of the pollen is dissolved and excreted. It seems likely that the fresh resin had a similar effect on some pollen, but the chemical processes that might have caused this effect during embedding or amberization are unknown.

## Conclusion

*Symplocos kowalewskii* (Symplocaceae) from Baltic amber is the by far largest flower inclusion known. Its in-situ pollen, combined with morphology of the corolla and androecium, indicates strong affinities to extant Asian species of *S.* subgen. *Symplocos.* The large size of the corolla and its basal fusion to a staminate ring likely indicates entomophilous pollination, as is known for some Asian Symplocaceae. *S. kowalewskii* was likely a constituent of mixed-angiosperm-conifer forests in the Baltic amber source area and supports its affinities to evergreen broadleaved and mixed mesophytic forests of present-day East and Southeast Asia.

## Material and methods

### Origin and age of the amber fossil

The amber specimen X4088 is currently housed in a historic amber collection of the Federal Institute for Geosciences and Natural Resources (Bundesanstalt für Geowissenschaften und Rohstoffe, BGR) in Berlin-Spandau (Germany). The majority of Baltic amber kept in such historic collections likely derive from open cast mines of the Samland Peninsular (Kaliningrad, Russia^16^ and references therein). The so-called Blue Earth layer yields the highest amounts of amber and is therefore mainly subjected to mining^87–89^. Baltic amber is also occasionally found along the Baltic Sea coast, mostly being eroded from the Blue Earth that is exposed along the coast of the Samland Peninsular^90^. Different age estimates of the Blue Earth layer and its amber have been suggested, ranging from an early Eocene^91^ to late Eocene age^89–91^. Recently, the Lutetian age^91^ estimate has been critically discussed, as methodological issues might have led to an age overestimation^92,93^. Furthermore, most recent studies that combine biostratigraphic and mineralogical analyses of the Baltic amber deposit support the late Eocene age of the Blue Earth layer (37.8–33.9 Ma^94^; see^15,16^ for a detailed discussion on the age of Baltic amber).

### Preparation, imaging and pollen analysis

The amber specimen X4088 was enclosed in a glass chamber, filled with a solution of an extant dammar resin (*Shorea*, Dipterocarpaceae) and covered with a cover slip. This kind of preparation is typically found in historical Baltic amber collections that were established in Königsberg (today Kaliningrad, Russia) during the late 19th to early twentieth century (^95^ and references therein). In X4088 the glass chamber limited viewing details of the flower inclusion and inhibited pollen extraction. Moreover, the glass chamber and the cover slip were partially fractured and the embedding medium showed several signs of deterioration, including yellowing and fissures (Fig. 1a,b). We heated the specimen gently for five minutes at 38 °C in a vacuum oven (VO200, Memmert), which liquefied the embedding medium and allowed us to remove the fractured parts of the cover slip. As the resin cooled and began to cure, the amber specimen was again heated at 38 °C for several minutes. Then, the viscous resin was scratched out of the glass chamber by using a wooden pick and a scalpel. The remaining thin layer of sticky Dammer resin was ground and polished away by using wet silicon carbide paper (manufacturer Struers; see^95^ for protocols). A wet leather cloth with a tooth paste suspension was used for polishing all amber surfaces.

The amber inclusion was studied under a binocular microscope (Stemi 508, Carl Zeiss), a dissecting microscope (StereoDiscovery V8, Carl Zeiss) and a light microscope (AxioScope A1 KMAT, Carl Zeiss). Image stacks were taken with digital cameras (Canon EOS 80D), that were installed on each microscope, and further processed into photomicrographic composites by applying HeliconFocus. Up to nine singular photomicrographic composites were merged with the Adobe Photoshop CS6 23.0.0 software to create overview images of Fig. 1d,e. The overview images of Fig. 1a–c were taken using a photo station and a digital camera (Sony ILCE 7RM3), equipped with a Sony FE 50 mm F2.8 Macro Lens and the computer software Imaging Edge Desktop.

Pollen was carefully scratched out of one anther and the surrounding amber with a scalpel. We placed the samples on carbon-covered Scanning electron microscopy (SEM) mounts and sputtered them with gold (6 nm coat thickness) using an Automatic Sputter Coater. The pollen sample was examined under an electron microprobe with a field emission cathode (JEOL JXA-8500F).

For comparing the amber specimen to other fossil flowers of *Symplocos*, we used descriptions in the literature (as given in Table 1). For *S. bureauana,* microscopic images of the holotype MNHN.F-2170.2 (Paleontology collection, Muséum national d’Histoire naturelle, Paris, France; MNHN) helped to estimate the size of the flower organs.

## Supplementary Information

Supplementary Information.

## Data Availability

The fossil specimen is part of the public collection of the Federal Institute for Geosciences and Natural Resources (Bundesanstalt für Geowissenschaften und Rohstoffe, BGR) in Berlin-Spandau (Germany). All data generated or analysed during this study are included in this published article.
