# Supplementary material for: The largest amber-preserved flower revisited
Source: Sci Rep. 2023 Jan 12;13:17. doi: 10.1038/s41598-022-24549-z (PMC9837116; doi:10.1038/s41598-022-24549-z)
Supplement: Supplementary file 1 — Supplementary Information. [file 41598_2022_24549_MOESM1_ESM.docx]

| **Supplementary Table S1:** Sizes of flower inclusions from amber, taken from the indicated literature. Centered numbers are average values, size ranges are provided in brackets. | | |
| --- | --- | --- |
| **Amber deposit** | **Flower size** | **References** |
| Baltic amber | (0.2–) 4.2 (–15) mm long × (0.8–) 2.3 (–6.5) mm wide; (2–) 5.6 (–11) mm in diameter | 1, 2 |
| Le Quesnoy amber, France | 2.5 mm in diameter | 3 |
| Dominican amber | (1.4–) 4 (–9) mm long × (0.4–) 1.4 (–3.3) mm wide; 20 mm in diameter | 4‒7 |
| Rovno amber, Ukraine | 5 mm long × 3 mm wide | 8 |
| Chiapas amber, Mexico | 2.5 mm long × 20 mm wide | 9 |
| New Jersey amber | 3.5 mm long (perianth size) | 10 |
| Burmese amber | (1–) 4 (–5.8) mm long × 5–5.2 mm wide; (0.8–) 2.6 (–5.1) mm in diameter | 11‒19 |

**References:**

1. Conwentz, H. Die Angiospermen des Bernsteins in *Die Flora des Bernsteins und ihre Beziehungen zur Flora der Tertiärformation und der Gegenwart, 2. Band* (eds Göppert, H. R. & Menge, A.) 1–144 (W. Engelmann, 1886).
2. Sadowski, E.-M., Schmidt, A. R., & Denk, T. Staminate inflorescences with *in situ* pollen from Eocene Baltic amber reveal high diversity in *Fagaceae* (oak family). *Willdenowia* **50**, 405–517 (2020).
3. Del Rio, C., Haevermans, T. & De Franceschi, D. First record of an Icacinaceae Miers fossil flower from Le Quesnoy (Ypresian, France) amber. *Sci. Rep.* **7,** 11099; 10.1038/s41598-017-11536-y (2017).
4. Chambers, K. L. & Poinar, G. O. Jr. A fossil flower of the genus *Protium* (Burseraceae) in mid-Tertiary amber from the Dominican Republic. *J. Bot. Res. Inst. Texas* **7**, 367‒373 (2013).
5. Poinar, G. O. Jr. & Chambers, K. L. Mimosoideae (Fabaceae) diversity and associates in mid-Tertiary Dominican amber. *J. Bot. Res. Inst. Texas* **10**, 121‒136 (2016).
6. Poinar, G. O. Jr., & Steeves, R. *Virola dominicana* sp. nov. (Myristicaceae) from Dominican amber. *Botany* **91**, 530‒534 (2013).
7. Poinar, G. & Struwe, L. An asterid flower from neotropical mid-Tertiary amber. *Nat. Plants* **2,** 16005; 10.1038/nplants.2016.5 (2016).
8. Sokoloff, D. D. *et al.* Staminate flower of *Prunus s. l*. (Rosaceae) from Eocene Rovno amber (Ukraine). *J. Plant. Res.* **131,** 925–943 (2018).
9. Calvillo-Canadell, L., Cevallos-Ferriz, S. R. S. & Rico-Arce, L. Miocene *Hymenaea* flowers preserved in amber from Simojovel de Allende, Chiapas, Mexico. *Rev. Palaeobot. Palynol.* **160**, 126‒134 (2010).
10. Gandolfo, M. A., Nixon, K. C., Crepet, W. L. & Grimaldi, D. A. A late Cretaceous fagalean inflorescence preserved in amber from New Jersey. *Am. J. Bot.* **105**, 1–12 (2018).
11. Crepet, W. L., Nixon, K. C., Grimaldi, D. & Riccio, M. A mosaic Lauralean flower from the Early Cretaceous of Myanmar. *Am. J. Bot.* **103**, 290–297 (2016).
12. Chambers, K. L., Poinar, G. O. Jr. & Buckley, R*. Tropidogyne*, a new genus of Early Cretaceous Eudicots (Angiospermae) from Burmese amber. *Novon* **20**, 23‒29 (2010).
13. Poinar, G. O. Jr. A mid-Cretaceous Lauraceae flower, *Cascolaurus burmitis* gen. et sp. nov., in Myanmar amber. *Cretac. Res.* **71**, 96‒101 (2017).
14. Poinar, G. O. Jr. & Chambers, K. L. *Palaeoanthella huangii* gen. and sp. nov., an early Cretaceous flower (Angiospermae) in Burmese amber. *SIDA* **21**, 2087‒2092 (2005).
15. Poinar, G. O. Jr. & Chambers, K. L. *Tropidogyne pentaptera,* sp. nov., a new mid-Cretaceous fossil angiosperm flower in Burmese amber. *Palaeodiversity* **10**, 135‒140 (2017).
16. Poinar, G. O. Jr., Chambers, K. L. & Buckley, R. *Eoëpigynia burmensis* gen. and sp. nov., an early Cretaceous eudicot flower (Angiospermae) in Burmese amber. *J. Bot. Res. Inst. Texas* 1, 91‒96 (2007).
17. Poinar, G. O. Jr., Chambers, K. L. & Buckley, R. An early Cretaceous angiosperm fossil of possible significance in rosid floral diversification. *J. Bot. Res. Inst. Texas* **2**, 1183‒1192 (2008).
18. Poinar, G. O. Jr., Chambers, K. L. & Wunderlich, J. *Micropetasos*, a new genus of angiosperms from mid-Cretaceous Burmese amber. *J. Bot. Res. Inst. Texas* **7**, 745-750 (2013).
19. Shi, C. *et al.* Fire-prone Rhamnaceae with South African affinities in Cretaceous Myanmar amber. *Nat. Plants* **8**, 125–135, 0.1038/s41477-021-01091-w (2022).
